# Sporting Careers After ICD Implantation in Elite Athletes

**DOI:** 10.3390/jcdd13020097

**Published:** 2026-02-17

**Authors:** Marco Vecchiato, Florian Egger, Stefano Palermi

**Affiliations:** 1 Sports and Exercise Medicine Division, Department of Medicine, University of Padova, 35128 Padova, Italy; 2 Sports Medicine and Cardiovascular Rehabilitation Unit, Noale, 30033 Venice, Italy; 3 Department of Theoretical and Applied Sciences, eCampus University, 22060 Novedrate, Italy; 4 Institute of Sports and Preventive Medicine, Saarland University, 66123 Saarbrücken, Germany; florian.egger@uni-saarland.de; 5 Department of Medicine and Surgery, UniCamillus-Saint Camillus International University of Health Sciences, 00187 Rome, Italy; stefano.palermi@unicamillus.org

**Keywords:** implantable cardioverter defibrillator, athletes, sports, return to play, sports eligibility, sports cardiology

## Abstract

Background: The use of implantable cardioverter defibrillators (ICDs) in elite athletes following sudden cardiac arrest (SCA) or the diagnosis of high-risk cardiac conditions presents a complex interplay of medical, psychological, and legal challenges. Despite evolving guidelines, data on clinical outcomes and return-to-sport (RTS) trajectories in elite athletes remain limited. Objective: To describe the clinical profiles, management strategies, and career outcomes of elite athletes who received ICDs. Methods: A retrospective multilingual media and literature search was performed up to January 2026 to identify elite athletes with ICDs. Inclusion criteria required evidence of professional or Olympic-level competition, confirmed ICD implantation, and sufficient clinical and career data. Cases were analyzed for demographics, underlying diagnosis, prevention type, post-ICD outcomes, and RTS status. Results: Thirty-seven elite athletes were identified (mean age 25.8 ± 4.3 years). The most common sport was football (*n* = 25). Hypertrophic cardiomyopathy, non-ischemic LV scar, and arrhythmogenic cardiomyopathy were the most frequent diagnoses, although 49% of etiologies remained unspecified. ICDs were implanted for secondary prevention in 70% of cases. Following ICD implantation, 25 athletes (68%) completed RTS, including 24 (65%) at the professional level. Among these, nine experienced shocks, and four ultimately discontinued competition. The sole fatality occurred in an athlete who had voluntarily explanted the ICD. Conclusions: A substantial proportion of elite athletes with ICDs successfully return to high-level sport, but clinical outcomes, risk tolerance, and legal frameworks remain variable. These findings support continued shifts towards personalized shared decision making and highlight the need for standardized, sport-specific RTS protocols, long-term registries, and psychosocial support in this population.

## 1. Introduction

The sudden collapse of a seemingly healthy athlete on the field is one of the most alarming and high-profile medical emergencies in sport. Although rare, sudden cardiac arrest (SCA) remains the leading cause of non-traumatic death in young athletes during exercise [1]. With advances in cardiopulmonary resuscitation, public access to defibrillation, and post-arrest care, survival rates have improved. However, survivors, particularly those engaged in competitive sports, often face complex decisions regarding long-term management, including the implantation of an implantable cardioverter defibrillator (ICD) for secondary prevention [2].

ICDs are life-saving devices that prevent fatal arrhythmias by delivering a shock to restore normal heart rhythm [3]. Yet, in elite athletes, ICD use raises multifaceted challenges that go beyond arrhythmia control. These include concerns about device integrity during high-impact activity, the risk of inappropriate shocks, and the psychological and professional implications [4].

Historically, international guidelines have been cautious, discouraging return-to-sport (RTS) in athletes with ICDs, particularly in sports with high cardiocirculatory demands and exposure to relevant contacts [2]. However, growing evidence from observational studies and athlete registries suggests that RTS may be feasible in selected individuals, prompting a shift towards more permissive, athlete-centered approaches [5]. Despite this evolution, key questions remain about safety, eligibility, and the long-term outcomes of elite athletes competing with an ICD.

Most available data focus on recreational athletes or general ICD recipients [6], leaving a major knowledge gap regarding those at the highest levels of competition where performance, public visibility, and legal obstacles intersect.

This work aims to bridge this gap by analyzing documented cases of elite athletes with ICDs regarding etiology, post-ICD outcomes and RTS. By synthesizing publicly available data, we aim to provide an updated understanding of how ICD implantation impacts athletic careers and long-term health outcomes, highlighting the need for standardized approaches and further research in this area.

## 2. Materials and Methods

### 2.1. Study Design

This study was designed as a retrospective, descriptive analysis aiming to identify and evaluate publicly documented cases of elite athletes who received an ICD. The objective was to systematically gather clinical and career outcome data in this unique population. As this study was based exclusively on publicly available data, institutional review board approval and informed consent were not required.

### 2.2. Search Strategy

To identify eligible cases, we performed two complementary search strategies.

First, a Boolean web-based search was conducted using Google in five languages (English, French, Spanish, German and Italian). The search strings were tailored to encompass all Olympic sports, ensuring broad inclusion across both individual and team disciplines (Supplementary Materials, Text S1). To supplement and expand case detection, a systematic media surveillance was performed using Meltwater (San Francisco, CA, USA), a global press monitoring platform scanning over 3.1 million articles daily across 252 countries. A dedicated physician (FE) conducted daily, reporter-independent media searches in four languages (English, French, Spanish, German) using predefined Boolean search terms (Supplementary Materials, Text S2).

Second, a systematic literature search was carried out on PubMed using a predefined set of medical subject headings (MeSH) and keywords related to athletes and implantable defibrillators (Supplementary Materials, Text S3). Searches were conducted from database inception until January 2026.

This approach was intentionally designed as a descriptive case series based on publicly available sources rather than a systematic review, and therefore aims to capture high-visibility, well-documented cases rather than estimate incidence or prevalence.

### 2.3. Inclusion Criteria

To be included in this study, cases were required to meet all of the following criteria:

- Level of competition: The athlete must have competed at a professional, Olympic, or elite national/international level. Professional competition was defined as participation under formal contract and/or salaried employment within a recognized top-tier league or federation. Elite status was defined as sustained involvement in top-division leagues, international championships, or official representation of a national team. This definition was applied consistently across all included cases and served as a mandatory eligibility criterion for study inclusion. Collegiate athletes were excluded unless they also met one of the above criteria. Reports lacking a clear classification of competitive level were excluded.
- Documentation of ICD implantation: ICD placement had to be explicitly verified through reputable media coverage or publicly available institutional statements.
- Data availability: Sufficient clinical and career information needed to be accessible for analysis, including the athlete’s age at implantation, year of implantation, clinical indication, and post-ICD career trajectory.

No restrictions were placed on the date of ICD implantation; all publicly available cases were eligible for inclusion. Athletes known to have suffered a SCA without confirmed ICD implantation were excluded.

### 2.4. Data Verification

All included cases were independently reviewed and verified by two physicians with expertise in sports cardiology (MV and SP). Any discrepancies in eligibility or extracted data were resolved through consensus with a third sport physician (FE). To enhance data accuracy, additional targeted web searches were performed for each athlete to corroborate and complete clinical and career-related information. Where the athlete’s identity or diagnosis was uncertain, inclusion required corroboration from at least two independent sources. Given the reliance on publicly available and media-reported data, particular attention was paid to internal consistency and clinical plausibility of the reported information. Cases with unclear, contradictory, or insufficient clinical details were excluded. This verification process was specifically implemented to mitigate the risk of misclassification and reporting bias inherent to media-based case identification.

## 3. Results

A total of 37 elite athletes with confirmed ICD implantation were identified and analyzed. All identified cases had verifiable data regarding the initial cardiac event, diagnosis, ICD management, and post-implantation outcomes.

Table 1 summarizes the demographic, diagnostic, and outcome data of the included athletes included (35 males and 2 females) in this study. Sports represented in this cohort included football (*n* = 19), ice hockey (*n* = 4), athletics (*n* = 3), cycling (*n* = 2), volleyball (*n* = 2), basketball (*n* = 2), futsal (*n* = 2), rugby (*n* = 1), and handball (*n* = 1). The mean age at the time of ICD implantation was 25.8 ± 4.3 years. Belgium was the most frequently represented country, accounting for four athletes.

No publicly available information was found regarding concomitant medication use. Implantation procedure details were reported in seven cases, including six subcutaneous ICDs and one epicardial ICD.

### 3.1. Diagnosis and Etiology

The most frequent underlying conditions were hypertrophic cardiomyopathy (HCM) in seven athletes and non-ischemic left ventricular scarring in five athletes. Arrhythmogenic right cardiomyopathy (ARVC) was identified in three cases, two athletes were identified having acute myocarditis while in two athletes the presence of an accessory pathway (WPW syndrome) was reported. In 18 athletes (49%), the etiology remained unclear or not publicly confirmed.

### 3.2. Post-ICD Outcomes

**Figure 1:**
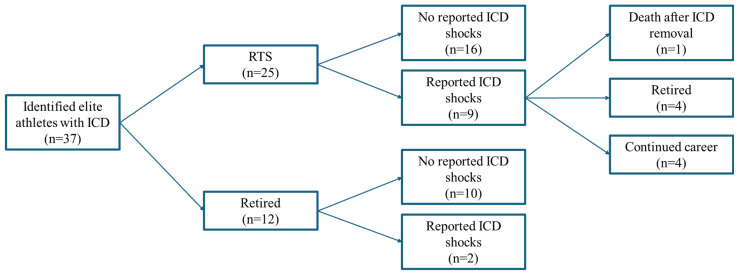
Flowchart of post-ICD career outcomes in 37 identified elite athletes.

Among the 37 included athletes (Figure 1):

- A total of 25 athletes (68%) returned to competitive sport. Of these, 24 resumed at the elite/professional level, while one returned to the amateur level (football). Nine experienced one or more ICD discharges. In four of these nine cases, recurrent episodes and/or medical advice ultimately led to restriction or cessation of athletic activity. One athlete died approximately 22 months after voluntarily explanting the previously implanted ICD.
- A total of 12 athletes (32%) permanently retired from competitive sport immediately after SCA without attempting a return to competitive activity. In two of these athletes, an ICD shock was documented: one occurred during an official match while serving as a coach, and the other during non-competitive physical activity.

### 3.3. Clinical Presentation

**Figure 2:**
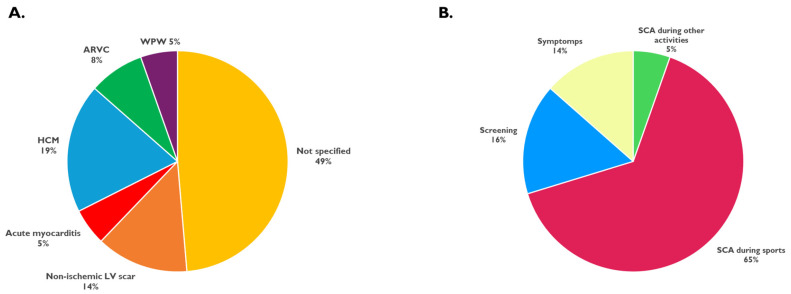
Clinical presentation of elite athletes receiving ICDs. (**A**). Cardiac diagnoses among elite athletes who received an ICD. (**B**). Clinical events leading to ICD implantation. ARVC: arrhythmogenic right cardiomyopathy; HCM: hypertrophic cardiomyopathy; SCA: sudden cardiac arrest; WPW: Wolff–Parkinson–White syndrome.

Among the 37 included athletes (Figure 2):

- Twenty-six athletes (70%) experienced a documented SCA with 24 being sports-related SCA during official competition or training, and two during non-sporting activities (while driving and during sleep, respectively). Two athletes who experienced SCA had previously received a medical diagnosis of a high-risk cardiac condition, identified through pre-participation screening, without undergone ICD implantation.
- Eleven athletes (30%) received an ICD without a prior SCA due to diagnosis of a high-risk proarrhythmic cardiac condition. Six athletes were identified during routine cardiac screening (two due to resting ECG abnormalities, two due to exercise-induced arrhythmias, and two for non-specified findings), while five were diagnosed due to symptoms (one for syncope, two for palpitations, two for exercise-induced dizziness).

### 3.4. Regulatory and Legal Considerations

Regulatory restrictions significantly influenced some athletes’ RTS decisions. Three athletes moved to different countries in order to continue competing after ICD implantation due to limitations imposed by national laws on sports eligibility.

## 4. Discussion

This case series describes a unique cohort of elite athletes who underwent ICD implantation for either primary or secondary prevention of SCA. By synthesizing publicly available data, we sought to describe clinical scenarios, diagnostic profiles, device management, and post-implantation outcomes in this population. Our findings reveal that approximately two-thirds of athletes were able to resume competitive sport, with the majority returning to the elite level. However, ICD discharges, recurrent symptoms, and medical or regulatory challenges led to subsequent withdrawal in several cases.

### 4.1. Epidemiological Considerations

Notably, only two female athletes were identified in this cohort, highlighting a marked gender disparity. While this may partially reflect the overrepresentation of males in elite-level sport, it is also consistent with data from international registries [7,8]. This imbalance may further be influenced by underdiagnosis, underreporting, or differential media coverage in female sports [9]. The emerging literature suggests that women may present with different arrhythmic symptoms, face longer diagnostic delays, and are less likely to be cleared for competitive sport following cardiac diagnoses [10].

Diagnostic heterogeneity was notable, with a significant proportion of cases remaining etiologically undefined since not in the public domain. The most frequently identified diagnoses were HCM, non-ischemic ventricular scarring and ARVC. However, in about half of cases the precise diagnosis remained unspecified or unconfirmed in public sources, which might be attributed to a certain underreporting as reported from earlier observational series [11,12].

About one third of the included athletes received a diagnosis of heart disease without a prior SCA detected through cardiac pre-participation screening (PPS). These included ECG abnormalities, exercise-induced arrhythmia, syncope, and palpitations. Indeed, most of these athletes received an ICD for primary prevention following the detection of a silent but potentially life-threatening condition during routine PPS, underscoring its important preventive role [13]. Although PPS protocols vary between countries, high-level athletes are typically screened with a combination of resting ECG, exercise testing (often cardiopulmonary), and transthoracic echocardiography [14,15,16]. These screening modalities allow for the detection of a wide range of asymptomatic cardiac abnormalities that might otherwise remain unnoticed until a catastrophic event occurs [17]. Furthermore, two additional athletes in this series had a documented history of potentially high-risk findings but were not fitted with an ICD until after the occurrence of a SCA.

While presumed PPS was in place for most athletes, 24 athletes still experienced a SCA without a prior diagnosis, highlighting the limitations of current screening protocols in detecting concealed arrhythmic substrates [18,19]. Although ECG has shown high accuracy in distinguishing pathological conditions from athletic heart adaptations, certain phenotypically silent or non-structural conditions may still elude detection. Artificial intelligence could play a role in enhancing such protocols in the next future [20,21].

While ARVC consistently led to disqualification, conditions such as HCM or post-inflammatory scarring were managed more variably, with some athletes returning to sport under close monitoring [22,23,24,25]. These differences stress the need for harmonized diagnostic criteria and risk stratification protocols following abnormal findings in asymptomatic athletes.

### 4.2. Return to Sport and Legal Barriers

RTS decisions were markedly variable. Of the 24 athletes who resumed sport, 10 experienced clinically significant symptoms or ICD discharges, and 7 eventually discontinued competition due to recurrent events. Notably, one athlete died after voluntary ICD explant, emphasizing the device’s life-saving role and the potential risks of premature withdrawal. These findings emphasize that RTS is not a static decision, but a dynamic process requiring longitudinal reassessment and active surveillance [26].

Shared decision making has emerged as a cornerstone in sports cardiology, particularly in the context of ICD carriers, where binary recommendations may not capture the athlete’s values, psychological readiness, or level of supervision available [27,28]. This paradigm is further challenged in countries with rigid restrictions on sport participation following ICD implantation, prompting some athletes to move to different countries in order to resume their career. These cases highlight the tension between legal standards and individualized risk management, raising important ethical and practical questions.

### 4.3. Focus on Sports

RTS with an ICD is not solely a question of arrhythmic risk but must also consider the potential for device-related trauma. Contact sports such as rugby, football, and ice hockey pose biomechanical threats to ICD integrity through direct blows to the chest, body collisions or repetitive upper-body motion. While only one device failure was reported in this series [29], long-term risk remains a concern, particularly with traditional transvenous systems. Protective strategies including precordial padding, surgical placement in less exposed areas, and the use of subcutaneous ICDs (S-ICD) should be part of the decision-making process. Sport-specific guidelines should explicitly address the mechanical risk for inappropriate shocks and other harms such as lead damage in direct context with the different implantation procedures and potential protective equipment with the aim to outline mitigation protocols for contact sports.

Football was the most common sport in this cohort (51%), not only reflecting its global prevalence but also the high visibility of cardiac events on the field. This subgroup illustrated the full range of outcomes: from successful RTS to retirement due to symptoms or legal restrictions. The cases of footballers provide compelling evidence for sport-specific RTS policies and highlight the influence of media, national and international football associations, and public opinion on clinical decisions.

Disparities were also observed across other contact sports with a high rate of bodily collisions and blows. While rugby players were occasionally cleared for RTS, all professional ice hockey players in this cohort retired after ICD implantation. These patterns mirror different decision-making processes despite similar sport-specific biomechanical risks, prompting more explicit eligibility criteria to be integrated into RTS recommendations.

### 4.4. Are Elite Athletes with ICD Different from the Other Athletes with ICD?

Compared with broader ICD populations, elite athletes appear to demonstrate higher rates of successful RTS, 62% in this series. While existing registries have focused on this subgroup [2], it is plausible that elite athletes benefit from superior physiological conditioning, closer medical surveillance, and higher intrinsic motivation. These factors are likely to contribute to enhanced resilience and higher RTS success.

The ICD Sports Safety Registry, the largest study on this topic, found no deaths, injuries, or major adverse events in athletes (not necessarily elite) competing with an ICD, including in high-intensity disciplines [30]. Subsequent analyses showed that competitive athletes were more likely to experience multiple appropriate ICD shocks compared to recreational athletes, though without fatal consequences [31]. This supports the notion that competitive intensity may increase arrhythmic risk but does not negate the protective effect of ICDs.

Exercise can increase the propensity of inappropriate shocks by one of two mechanisms: (1) sinus tachycardia rates can compete with therapy zones and trigger inappropriate therapy and/or (2) T wave changes during exercise can lead to “double-counting” of sinus tachycardia leading to inappropriate therapy. Both issues can be avoided with age and disease-specific device programming and sophisticated sensing algorithms. Modern device programming (e.g., higher detection thresholds, longer detection intervals) has further reduced inappropriate shocks, as demonstrated by several trials. Recent data also validate the safety of S-ICDs in athletic populations, with a caveat of increased early postoperative infection risk [32]. These findings have led to a paradigm shift—from rigid disqualification policies to individualized, shared decision-making frameworks.

Most recent international guidelines emphasize tailoring RTS decisions to the athlete’s condition, sport, and preferences [2,33]. However, inconsistent implementation across jurisdictions continues to challenge equitable access to sport.

Although device type was specified in only a minority of cases, the presence of S-ICDs and epicardial systems in selected athletes warrants further experience. The choice of ICD configuration has significant implications for athletes, particularly those involved in contact or high-impact sports [34]. Transvenous systems, while traditional, carry long-term risks of lead fracture or venous occlusion. While S-ICD eliminates the risk of transvenous lead-related complications, their performance in high-contact sports remains under evaluation. Although some data suggest they may offer a favorable profile in these contexts by avoiding vascular structures, current evidence is insufficient to definitively favor S-ICDs over traditional systems in elite contact athletes [33,35]. The limited reporting of device type in public sources underscores the need for transparent documentation and consideration of device selection in future RTS frameworks.

### 4.5. Long-Term Outcomes and Psychological Impact

The psychological burden of living and competing with an ICD is profound. Athletes frequently report fear of shocks, anxiety about performance limitations, and existential stress over career disruption. Moreover, arrhythmic risk extends beyond competition and training, and athletes should be adequately informed that ICD discharges may also occur during daily life activities. For many, sport is both identity and livelihood. Career-ending cardiac diagnoses and living with ICD can lead to emotional distress and socioeconomic instability [6,25].

High-profile cases like the professional football player Daley Blind have publicly described the vulnerability of competing with an ICD and the adjustment required to regain confidence. These narratives highlight the critical need for integrated psychological support in the post-implantation care pathway.

### 4.6. Importance of Early Recognition, AED Access, and Education

The fact that the majority of ICD implantations followed a SCA, many of which occurred during sport, underscores the importance of emergency preparedness. Timely cardiopulmonary resuscitation and automated external defibrillator (AED) access are critical determinants of survival and favorable neurological outcomes [36]. The visibility of these events in professional sport offers a powerful platform to advocate for widespread AED availability, athlete education, and emergency planning. Sport settings, due to the physical stress involved and the young demographic of participants, should be considered high-priority environments for the deployment of AEDs [37]. It is therefore essential to not only equip sports venues with AEDs but also to develop a robust culture of readiness through structured training, periodic retraining, and removal of perceived legal risks for responders [36]. Educational campaigns targeting athletes, coaches, and the public can further bridge the gap between legislation and practice. Importantly, beyond their individual clinical trajectories, several elite athletes with ICDs have assumed an active role as advocates for cardiac screening, CPR training, and SCA awareness in sports. Their lived experience provides a unique and credible perspective that can positively influence athletes, sporting organizations, and the general public. From a policy perspective, involving these athletes in structured prevention and screening initiatives may represent an effective strategy to promote cardiovascular safety in sport.

### 4.7. Methodological Considerations

This study has several important limitations. First, the case series is based on publicly available data and published case reports, which may introduce selection and reporting biases. Athletes with more severe outcomes or higher public visibility are more likely to be documented, potentially overestimating the rate of RTS or the frequency of recurrent events. Clinical details such as genetic testing results, precise ICD programming, or follow-up duration were often missing or inconsistently reported, limiting the ability to draw general medical conclusions. Moreover, although the media surveillance platform was not subject to language restrictions, the web search was language-limited (automated translation tools were still used) for practical feasibility reasons; therefore, language-related constraints may still have limited the number of cases identified. Second, the classification of diagnoses and outcomes relied on the best available information, but may be subject to misclassification or interpretation bias, particularly in cases labeled as “unspecified etiology”. Lastly, differences in national regulations, medical cultures, and access to sports cardiology expertise may have influenced decision making across cases, limiting the generalizability of the findings to broader populations.

Additionally, the reliance on public and media-reported data introduces a potential bias related to athlete visibility and sociocultural factors [38,39]. High-profile sports like football are more likely to be covered by international media, whereas athletes from lower-tier leagues, less popular sports, or non-Western countries may remain undocumented. Cultural stigma, legal ramifications, and differing media landscapes may also affect the willingness to disclose a diagnosis or ICD implantation. These factors are likely to contribute to geographic and sport-specific clustering in our sample, limiting its representativeness despite a broad multilingual search.

While limitations exist, this study provides valuable insight and represents a notably comprehensive overview of ICD outcomes in elite athletes to date. This study underscores the urgent need for multicenter registries and longitudinal studies to better understand the implications of ICDs in elite athletes. Standardizing RTS protocols and fostering collaboration among cardiologists, sports physicians, and regulatory bodies will be pivotal. Furthermore, the integration of shared decision-making models can empower athletes while ensuring safety.

## 5. Conclusions

The implantation of an ICD in elite athletes represents a medical, ethical, and regulatory crossroads. While ICDs are unequivocally life-saving, their impact on the careers and health trajectories of athletes is multifaceted. This study sheds light on the diversity of outcomes among elite athletes who received ICDs and highlights the critical role of legal and medical frameworks. By adopting standardized approaches and leveraging data-driven insights, the sports and medical communities can better support athletes in navigating these challenges.

## Figures and Tables

**Table 1:** Identified elite athletes with ICDs. Demographic data, clinical scenarios, diagnostic profiles, device management, and post-implantation outcomes of 37 elite athletes fitted with ICD.

ID#	Sex	Sport	Nation	Age at Implantation	Year of ICD Implantation	Cardiac Disease	Circumstances	Post-ICD Career Outcome
1	M	Football	Denmark	29	2021	Not specified	Collapsed during a match in June 2021; successfully resuscitated and received an ICD.	Returned to professional football in early 2022; continues to play at a competitive level, including appearances for the national team. Transferred to another country due to national law regulations.
2	M	Football	Netherlands	29	2019	Myocarditis	Experienced dizziness during a match in December 2019; diagnosed with myocarditis and received a subcutaneous ICD.	Returned to professional football in early 2020; continues to play at a competitive level, including appearances for the national team. In August 2020, ICD activation during a friendly match; no further major incidents reported.
3	M	Football	UK	23	2012	HCM	SCA during a match in March 2012; heart stopped for 78 min; successfully resuscitated and received an ICD.	Retired due to persistent arrhythmias in August 2012 following medical advice.
4	M	Football	Germany	24	2013	Myocarditis	SCA during a match in July 2013; diagnosed with myocarditis and chronic arrhythmia. Underwent four heart surgeries, including ICD implantation.	Returned to professional football in November 2014, becoming the first professional player in Germany to play with an ICD. Continued playing professionally until 2017; retired in 2018 after experiencing multiple ICD shocks. Serves as a public ambassador for SCA awareness and prevention.
5	M	Football	Belgium	21	2008	HCM	Diagnosed with cardiac arrhythmia in 2008; received an ICD. In June 2009, the ICD successfully intervened during a match after he collapsed on the field. In May 2018, experienced another collapse during a game; ICD activated.	Continued professional football career post-ICD implantation until November 2018, when he retired due to recurrent cardiac events.
6	M	Football	Ghana	24	2018	Not specified	Diagnosed with unspecified heart condition in 2017; underwent ICD implantation in January 2020.	Continued professional football career despite medical advice to retire. Collapsed during a match in October 2021; ICD intervened successfully. In 2022, his ICD was reportedly removed. On 11 November 2023, suffered SCA during a match and was pronounced dead upon arrival at the hospital.
7	M	Football	Wales	28	2023	Accessory pathway—WPW	Collapsed during a match in May 2023 due to atrial fibrillation; underwent corrective surgery. In December 2023, suffered a SCA during a Premier League match; successfully resuscitated and received an ICD.	Returned to competitive football at lower professional level, following individualized medical clearance and rehabilitation.
8	M	Football	UK	28	2021	Not specified	Suffered SCA during training in November 2021; successfully resuscitated and received ICD implantation.	Returned to professional football in 2022; continues to play at a competitive level without further reported events. In March 2022, experienced another cardiac event during training, with successful ICD intervention.
9	M	Football	Serbia	25	2023	Not specified	Suffered SCA during a match in March 2023; successfully resuscitated and underwent ICD implantation.	Experienced a second collapse during play in December 2023. Returned to professional football after ICD implantation but permanently retired in July 2024 following recurrent collapse and medical advice.
10	M	Football	Senegal	28	2004	Not specified	Diagnosed with unspecified heart condition in 2003; did not make any official appearances for the club. In October 2004, collapsed during warm-up before a match; subsequently received an ICD.	Returned to professional football post-ICD implantation, playing for various clubs. Continued his career until retiring in 2011.
11	M	Football	Senegal	20	2024	Myocarditis-induced ventricular arrhythmia with myocardial scarring	Diagnosed with heart rhythm irregularities in 2023; contract terminated due to national policies. In November 2024, collapsed during a match; diagnosed with arrhythmia and received an ICD.	In 2025 returned to professional football; no official retirement announced.
12	M	Football	Netherlands	23	2010	Not specified	SCA during a match in September 2010; successfully resuscitated and received an ICD.	Continued professional football career post-ICD implantation. In September 2012, experienced another cardiac arrest during a match; ICD intervened successfully. Retired in 2018 after playing for various clubs.
13	M	Football	Italy	21	2024	Non-ischemic LV scar	SCA during a match in December 2024; successfully resuscitated and received an ICD.	Returned to professional football in early 2026. Transferred to another country due to national law regulations.
14	M	Cycling	Italy	31	2022	Not specified	SCA after finishing second in a race in March 2022; successfully resuscitated and received an ICD.	Retired from professional cycling in October 2022 due to medical advice and national regulations prohibiting competition with an ICD.
15	M	Cycling	Belgium	27	2023	Not specified heart wall disease.	SCA while driving, leading to a multi-car accident in September 2023; successfully resuscitated and hospitalized. Subsequent tests revealed a heart muscle anomaly causing arrhythmia and received an ICD implantation.	Retired from professional cycling in September 2023 following ICD implantation
16	M	Athletics	Jamaica	28	2019	Not specified	SCA while pacing the men’s 3000 m race on February 2019; successfully resuscitated and received an ICD.	Retired from professional running in September 2019 following medical advice; currently involved in coaching and raising awareness about heart health.
17	F	Athletics	Germany	28	2019	Unspecified congenital cardiac arrhythmia	Diagnosed with congenital cardiac arrhythmia, she experienced episodes of abnormally fast heartbeats from a young age. In October 2018, she received a subcutaneous ICD to manage her condition.	Continued her professional pole-vaulting career post-ICD implantation, including participation in the Olympics. As of 2025, she remains active in athletics and serves as an advocate for heart rhythm awareness.
18	F	Handball	Danmark	30	1999	Not specified	SCA on court in 1999. Received an ICD implantation. Collapsed again in 2003 while coaching a Champions League match.	Retired from professional handball; transitioned to a successful coaching career.
19	M	Ice Hockey	Canada	23	2012	Not specified	SCA during a recreational hockey game in July 2012; successfully resuscitated and received an ICD.	Retired from professional hockey. Subsequently became an advocate for CPR and AED awareness, collaborating with organizations like the Heart and Stroke Foundation.
20	M	Ice Hockey	Canada	26	2016	Not specified	SCA during pre-game warm-up in November 2016. Resuscitated using extracorporeal membrane oxygenation and underwent emergency surgery. Due to complications, underwent left leg amputation below the knee.	Retired from professional hockey. Transitioned to a scouting role. Founded the All Heart Foundation to promote cardiac screening and awareness.
21	M	Ice Hockey	Canada	31	2014	Accessory pathway—WPW	SCA on the bench during an NHL game in March 2014; successfully resuscitated, underwent ablation and received an ICD.	Retired from professional hockey in September 2015. Transitioned to a player development role. Founded an initiative to promote heart health awareness and AED accessibility.
22	M	Volleyball	Belgium	19	2022	Not specified	SCA during a match in October 2022; resuscitated and received an ICD.	In February 2024, collapsed again during a Belgian league match; ICD successfully intervened. Announced retirement from professional volleyball in February 2024 following medical advice after the second cardiac event.
23	M	Volleyball	Belgium	33	2013	Not specified	SCA during a training session in August 2009 and was placed in an induced coma. He received an ICD following the incident.	Transferred to another country due to national law regulations. New SCA in 2013 during a match; ICD successfully intervened. Retired from volleyball in 2019.
24	M	Rugby	Wales	22	2023	Not specified	SCA during sleep in August 2023; resuscitated after 17 min. Received an S-ICD.	Returned to rugby; became an advocate for CPR and heart health awareness.
25	M	Ice Hockey	Canada	36	2020	Not specified	SCA on the bench during an NHL game in February 2020; revived with CPR and AED. Received an ICD.	Retired from professional hockey in January 2021; no return to competition.
26	M	Football	Denmark	33	2023	Apical HCM with fibrosis and fat infiltration	Experienced dizziness during training in May 2023; diagnosed with a heart condition and received an ICD.	Returned to competitive play in April 2024 after an 11-month hiatus.
27	M	Football	Germany (likely)	23	2015 (likely)	HCM (mid-ventricular/apical)	Diagnosed during evaluation for abnormal ECG; ICD implanted for primary prevention.	Returned to professional football with close monitoring and individualized clearance.
28	M	N/A	USA	21	N/A (2021–2022)	Apical HCM (MYBPC3 mutation)	Diagnosed after abnormal ECG at age 17; progressive hypertrophy and LGE at CMR led to ICD implantation.	Returned to professional sport with ICD; remains asymptomatic under close follow-up.
29	M	Basketball	Italy	21	N/A (2017–2018)	ARVC (PKP2 mutation + variant in RYR2)	VF during a match treated with CPR and 7 external shocks; S-ICD implanted for secondary prevention.	Disqualified from competitive sports. One year later, experienced VT during basketball activity, successfully terminated by S-ICD activation.
30	M	Cycling	Italy	20	N/A (2018 likely)	ARVC	Palpitations, presyncope, and documented NSVT during cycling competition; diagnosed based on imaging and biopsy. S-ICD implanted for primary prevention.	Disqualified from competitive sports.
31	M	Football	Sweden (likely)	30	N/A (2024 likely)	Apical HCM with aneurysm, fibrosis and fat infiltration	Syncope during training in early 2024; prior abnormal ECG since adolescence. ICD implanted following shared decision making.	Returned to professional football after 7-month encapsulated rehabilitation; continues under close cardiac monitoring.
32	M	Football	England/Nigeria	22	2022	Non-ischemic LV scar	Diagnosed in early 2021 during medicals checks with myocarditis followed by fibrotic scar; S-ICD implanted in September 2022.	Returned to professional football in March 2024 after three-year hiatus.
33	M	Basketball	USA	N/A	2017	HCM	Diagnosed in 2018 during pre-college screening; ICD implanted subsequently.	Continues professional basketball career in the NBA under regular cardiac monitoring.
34	M	Football	Jordan	23	2022	ARVC	SCA during warm up in 2022. VF for 15 min, converted after 7 shocks; ICD implanted subsequently. No permanent complications.	Retired. No further details.
35	M	Football	France/Algeria	29	2024	Non-ischemic LV scar	SCA during a match in June 2024. Reanimated with AED by his teammates and received ICD. He received regular screening until May 2024. Only a sequalae on MRI.	Returned to professional football in February 2025 and scored in his comeback match.
36	M	Futsal	New Zealand	24	2017	Not identified	Collapsed during futsal training. CPR by player who was a doctor and reanimated with AED. 3 days ICU and intensive investigations without finding a cause. ICD implanted.	Returned to competitive futsal and football.
37	M	Futsal	Australia	30	2016	Not specified	SCA immediately after coming off the field. CPR by paramedics and AED discharge efficiently. ICD was implanted.	Returned to competitive futsal.

AED: Automated External Defibrillator; ARVC: Arrhythmogenic Right Ventricular Cardiomyopathy; CMR: Cardiovascular Magnetic Resonance; CPR: Cardiopulmonary Resuscitation; ECG: Electrocardiogram; HCM: Hypertrophic Cardiomyopathy; ICD: Implantable Cardioverter Defibrillator; ICU: Intensive Care Unit; LGE: Late Gadolinium Enhancement; LV: Left Ventricle; MRI: Magnetic Resonance Imaging; N/A: Not Available or Not Applicable; NBA: National Basketball Association; NHL: National Hockey League; NSVT: Non-Sustained Ventricular Tachycardia; PKP2: Plakophilin-2 gene; RYR2: Ryanodine Receptor 2 gene; S-ICD: Subcutaneous Implantable Cardioverter Defibrillator; SCA: Sudden Cardiac Arrest; UK: United Kingdom; USA: United States of America; VF: Ventricular Fibrillation; VT: Ventricular Tachycardia; WPW: Wolff–Parkinson–White syndrome.

## Data Availability

The data supporting the findings of this study are available from the corresponding author upon reasonable request.

## Supplementary Materials

The following supporting information can be downloaded at: https://www.mdpi.com/article/10.3390/jcdd13020097/s1, Text S1: Search string in five languages used with Google. Text S2: Search string used with Meltwater monitoring software. Text S3: Search string used with PubMed database.
