# Supplementary material for: Sporting Careers After ICD Implantation in Elite Athletes
Source: J Cardiovasc Dev Dis. 2026 Feb 17;13(2):97. doi: 10.3390/jcdd13020097 (PMC12941241; doi:10.3390/jcdd13020097)
Supplement: Supplementary file 1 [file jcdd-13-00097-s001.zip › jcdd-4137813-supplementary.pdf]

ST1. Search string in five languages used with Google.

English Search String

"athlete" AND ("implantable cardioverter defibrillator" OR "ICD") AND ("professional sports" OR "soccer" OR "basketball" OR "volleyball" OR "ice hockey" OR "athletics" OR "golf" OR "swimming" OR "tennis" OR "rugby" OR "cycling" OR "triathlon" OR "marathon" OR "boxing" OR "MMA" OR "wrestling" OR "cricket" OR "baseball" OR "softball" OR "rowing" OR "canoeing" OR "kayaking" OR "fencing" OR "archery" OR "skiing" OR "snowboarding" OR "speed skating" OR "figure skating" OR "bobsleigh" OR "skeleton" OR "luge" OR "surfing" OR "skateboarding" OR "equestrian" OR "climbing" OR "badminton" OR "squash" OR "handball" OR "water polo" OR "field hockey" OR "lacrosse" OR "diving" OR "sailing" OR "judo" OR "taekwondo" OR "karate" OR "weightlifting" OR "gymnastics" OR "pentathlon" OR "wushu" OR "track cycling" OR "mountain biking" OR "cross-country skiing")

French search string

"athlète" AND ("défibrillateur cardiaque implantable" OR "ICD") AND ("sports professionnels" OR "football" OR "basket-ball" OR "volley-ball" OR "hockey sur glace" OR " athlétisme" OR "golf" OR "natation" OR "tennis" OR "rugby" OR "cyclisme" OR "triathlon" OR "marathon" OR "boxe" OR "MMA" OR "lutte" OR "cricket" OR "baseball" OR "softball" OR "aviron" OR "kayak" OR "escrime" OR "tir à l'arc" OR "ski" OR "snowboard" OR "patinage de vitesse" OR "patinage artistique" OR "bobsleigh" OR "skeleton" OR "luge" OR "surf" OR "skateboard" OR "équitation" OR "escalade" OR "badminton" OR "squash" OR "handball" OR "water-polo" OR "hockey sur gazon" OR "lacrosse" OR "plongeon" OR "voile" OR "judo" OR "taekwondo" OR "karaté" OR " haltérophilie" OR "gymnastique" OR "pentathlon" OR "wushu" OR "cyclisme sur piste" OR "VTT" OR "ski de fond")

Spanish search string

"atleta" AND ("desfibrilador automático implantable" OR "ICD") AND ("deportes profesionales" OR "fútbol" OR "baloncesto" OR "voleibol" OR "hockey sobre hielo" OR "atletismo" OR "golf" OR "natación" OR "tenis" OR "rugby" OR "ciclismo" OR "triatlón" OR "maratón" OR "boxeo" OR "MMA" OR "lucha libre" OR "críquet" OR "béisbol" OR "softbol" OR "remo" OR "piragua" OR "esgrima" OR "tiro con arco" OR "esquí" OR "snowboard" OR "patinaje de velocidad" OR "patinaje artístico" OR "bobsleigh" OR "skeleton" OR "luge" OR "surf" OR "monopatín" OR "equitación" OR "escalada" OR "bádminton" OR "squash" OR "balonmano" OR "waterpolo" OR "hockey sobre césped" OR "lacrosse" OR "clavados" OR "vela" OR "judo" OR "taekwondo" OR "karate" OR "halterofilia" OR "gimnasia" OR "pentatlón" OR "wushu" OR "ciclismo en pista" OR "bicicleta de montaña" OR "esquí de fondo")

German search string

"athlet" AND ("implantierbarer Kardioverter-Defibrillator" OR "ICD") AND ("Profisport" OR "Fußball" OR "Basketball" OR "Volleyball" OR "Eishockey" OR "Leichtathletik" OR "Golf" OR "Schwimmen" OR "Tennis" OR "Rugby" OR "Radfahren" OR "Triathlon" OR "Marathon" OR "Boxen" OR "MMA" OR "Ringen" OR "Cricket" OR "Baseball" OR "Softball" OR "Rudern" OR "Kanufahren" OR "Fechten" OR "Bogenschießen" OR "Skifahren" OR "Snowboarden" OR "Eisschnelllauf" OR "Eiskunstlauf" OR "Bobsport" OR "Skeleton" OR "Rodeln" OR "Surfen" OR "Skateboarden" OR "Reiten" OR "Klettern" OR "Badminton" OR "Squash" OR "Handball" OR "Wasserball" OR "Feldhockey" OR "Lacrosse" OR "Tauchen" OR "Segeln" OR "Judo" OR

"Taekwondo" OR "Karate" OR "Gewichtheben" OR "Turnen" OR "Moderner Fünfkampf" OR "Wushu" OR "Bahnradfahren" OR "Mountainbiken" OR "Langlauf")

Italian search string

"atleta" AND ("defibrillatore cardiaco impiantabile" OR "ICD") AND ("sport professionistici" OR "calcio" OR "basket" OR "pallavolo" OR "hockey su ghiaccio" OR "atletica leggera" OR "golf" OR "nuoto" OR "tennis" OR "rugby" OR "ciclismo" OR "triathlon" OR "maratona" OR "pugilato" OR "MMA" OR "lotta" OR "cricket" OR "baseball" OR "softball" OR "canottaggio" OR "canoa" OR "scherma" OR "tiro con l'arco" OR "sci" OR "snowboard" OR "pattinaggio di velocità" OR "pattinaggio artistico" OR "bob" OR "skeleton" OR "slittino" OR "surf" OR "skateboard" OR "equitazione" OR "arrampicata" OR "badminton" OR "squash" OR "pallamano" OR "pallanuoto" OR "hockey su prato" OR "lacrosse" OR "tuffi" OR "vela" OR "judo" OR "taekwondo" OR "karate" OR "sollevamento pesi" OR "ginnastica" OR "pentathlon moderno" OR "wushu" OR "ciclismo su pista" OR "mountain bike" OR "sci di fondo")

ST2. Search string used with Meltwater monitoring software.

(((athlete) near ("implantable cardioverter defibrillator" OR "ICD" OR "professional sports" OR "soccer" OR "basketball" OR "volleyball" OR "ice hockey" OR "athletics" OR "golf" OR "swimming" OR "tennis" OR "rugby" OR "cycling" OR "triathlon" OR "marathon" OR "boxing" OR "MMA" OR "wrestling" OR "cricket" OR "baseball" OR "softball" OR "rowing" OR "canoeing" OR "kayaking" OR "fencing" OR "archery" OR "skiing" OR "snowboarding" OR "speed skating" OR "figure skating" OR "bobsleigh" OR "skeleton" OR "luge" OR "surfing" OR "skateboarding" OR "equestrian" OR "climbing" OR "badminton" OR "squash" OR "handball" OR "water polo" OR "field hockey" OR "lacrosse" OR "diving" OR "sailing" OR "judo" OR "taekwondo" OR "karate" OR "weightlifting" OR "gymnastics" OR "pentathlon" OR "wushu" OR "track cycling" OR "mountain biking" OR "cross-country skiing")))

ST3. Search string used with Pubmed database.

(("Athletes"[Mesh] OR "Sports"[Mesh]) AND ("Defibrillators"[Mesh] OR "Defibrillators, Implantable"[Mesh] OR ”ICD”)).
